# Datasets on bulk density and coarse fragment content from the French soil quality monitoring network

**DOI:** 10.1016/j.dib.2024.110767

**Published:** 2024-07-24

**Authors:** Jose-Luis Munera-Echeverri, Line Boulonne, Nicolas P.A. Saby, Dominique Arrouays, Benoît Bertouy, Eva Lacarce, Floriane Serré, Benoit Toutain, Florent Millet, Thomas Loiseau, Manuel Martin

**Affiliations:** INRAE, Info&Sols,^1^ 45075 Orléans, France

**Keywords:** Soil monitoring networks, National scale, Soil organic carbon stocks, Available water capacity, Soil compaction

## Abstract

Various stakeholders, such as modelers, policy makers, farmers, and environmental regulators need reliable soil bulk density and coarse fragment content data. These two soil parameters are necessary to calculate soil carbon and nutrients stocks, to estimate water availability for plants, or to assess soil compaction. However, measuring these two parameters is labor intensive and time consuming. Therefore, many agricultural and environmental studies often miss these two soil parameters. Here, we provide four datasets, one with bulk density and coarse fragment contents of topsoil and subsoil, measured in two campaigns of the French Soil Quality Monitoring Network (RMQS for its acronym in French), a second one with the average values for bulk density and coarse fragments of the two campaigns at 0–30 cm and 30–50 cm. The third and the fourth ones are the raw data needed to calculate the two first datasets divided by campaign. In addition, the R script for calculating the depth-weighted values per soil layer is provided

Specifications Table

**Table:** 

Subject	Earth-Surface Processes
Specific subject area	Soil organic carbon stocks
Data format	Raw R scripts
Type of data	Tables
Data collection	As part of the French Soil Quality Monitoring Network (RMQS; Réseau de Mesure de la Qualité des Sols in French), two sampling campaigns for bulk density and coarse fragment content measurements were conducted in soil profiles distributed in a systematic sampling grid covering the French mainland territory. The samples were collected mainly by the ring and the excavation methods (a few sites were measured by alternative methods). In both campaigns, six samples were distributed down to 50 cm. Moreover, three other samples were taken from 50 cm down to 1 m depth only during the second campaign. The samples were dried in oven at 105 °C, and soil mass, fine earth, and coarse fragment content were measured.
Data source location	• Institution: INRAE • City/Town/Region: Orléans, Val de Loire. • Country: France.
Data accessibility	Repository name: GisSol Dataverse https://entrepot.recherche.data.gouv.fr/dataverse/gissol Four distinct datasets have been made available, each with a unique digital object identifier (DOI). Each dataset is accompanied by its unique access link, through which the data can be retrieved. The R script of the function to calculate the weighted means is shared within a version control system. **1. Raw data** **First campaign (Raw_data_RMQS_1):** Dataset name: Raw bulk density and coarse fragment data of the first campaign of the French Soil Quality Monitoring Network. Data identification number: https://doi.org/10.57745/7Y3G5W Direct URL to data: https://entrepot.recherche.data.gouv.fr/dataset.xhtml?persistentId=doi:10.57745/7Y3G5W
	Instructions for accessing these data: This dataset, accessible through a DOI, contains all the physicochemical analysis of the first campaign of RMQS. The containing file is called “RMQS1_bulk_density_raw_data.tab“ and the metadata file “RMQS1_metadata_bulk_density_raw_data.tab ”. In addition, a readme file is included (file readme.txt) with the specific information of this dataset. **2. Second campaign (Raw_data_RMQS_2):** Dataset name: Raw bulk density and coarse fragment data of the second campaign of the French Soil Quality Monitoring Network Data identification number: https://doi.org/10.57745/PYBCO9 Direct URL to data: https://entrepot.recherche.data.gouv.fr/dataset.xhtml?persistentId=doi:10.57745/PYBCO9 Instructions for accessing these data: The dataset, along with its accompanying metadata and readme file, are available for access. The dataset itself can be found in the file named “Raw_data_rmqs_2.tab”. The corresponding metadata can be found in the file “Metadata_raw_data_RMQS_2.tab”. Additionally, a readme file, named “readme_raw_data_rmqs2.txt”, provides further information and guidance regarding the dataset. **3. Dataset_A** Dataset name: Weighted average of bulk density, coarse fragment by layer of the first and second campaign of the French Soil Quality Monitoring Network. Data identification number: https://doi.org/10.57745/NHINJR Direct URL to data: https://entrepot.recherche.data.gouv.fr/dataset.xhtml?persistentId=doi:10.57745/NHINJR Instructions for accessing these data: The dataset can be found in the file “Dataset_A.tab”, the metadata in “Metadata_Dataset_A.tab”, and the readme file with the specific information of the dataset in “readme_datasetA.txt”. **4. Dataset_B** Dataset name: Data identification number: https://doi.org/10.57745/YVXHSI Direct URL to data: https://entrepot.recherche.data.gouv.fr/dataset.xhtml?persistentId=doi:10.57745/YVXHSI Average values of bulk density and coarse fragment at 0–30 cm and 30–50 cm of the first and second campaigns of the French Soil Quality Monitoring Network. Instructions for accessing these data: The dataset can be found in the file “Dataset_B.tab”, the metadata in “Metadata_Dataset_B.tab”, and the readme file in “readme_datasetB.txt”. **5. R Scripts** Git project name: BD_CF_RMQS Direct URL to data: https://forgemia.inra.fr/josemunera/bd_cf_rmqs Function for calculation of weighted average can be found in the R folder: R/weighted_average.R
Related research article	

## 1 Value of the Data

• Soil physical parameters such as bulk density (BD) and coarse fragment (CF) content are important in soil science because they are needed to calculate stocks, for example soil organic carbon in the context of climate change and the assessment of soils capacity to mitigate carbon dioxide emissions; trace metals in the context of soil pollution assessment, or nutrient stocks [[1], [2], [3]].
• Bulk density and CF content data are also useful to estimate available water capacity for plants using various pedotransfer functions [4]. Bulk density is also often used as an indicator of soil compaction [5], whereas CF may be useful to assess limitations to soil tillage [6], or to cultivate some crops, such as potatoes, carrots, beetroots, etc. [7].
• Measuring these two parameters is labor intensive and time consuming [8]. Soil monitoring programs very often miss these two soil physical parameters [9].
• Currently, gathering data on these two parameters has become increasingly important due to the need of calculation of soil organic carbon (SOC) or nutrient stocks, of carbon and nutrients balances and fluxes, and of relevant indicators for environmental policies.
• Therefore, there is an increasing demand of reliable data for both BD and CF contents from various stakeholders, such as modelers, policy makers, farmers, and environmental regulators.

## 2 Background

Soil BD is defined as the ratio between the dry mass of soil and its volume. BD is usually expressed in g cm^−3^ or Mg m^−3^. There are various methods for measuring BD. The cylinder and the excavation methods are among the most commonly used. The selection of a technique for a specific type of soil depends on the occurrence of CF and root density. The cylinder method is advisable for soils characterized by a low CF content and low root density, whereas the excavation method is employed in soils with a high CF content and a substantial presence of thick roots [8]. The CF content is defined as the soil fraction with a size larger than 2 mm [10]. While the fraction smaller than 2 mm is known as the fine earth fraction where SOC is stored [3]. The CF fraction may include roots larger than 2 mm but these are not considered as SOC but as plant biomass instead [11]. Often, the largest part of CF is formed by mineral coarse fragments which are considered to be free of organic carbon. Thus, correcting soil BD for the CF content is important to avoid overestimations of SOC stocks [11].

## 3 Data Description

**Fig. 1:**
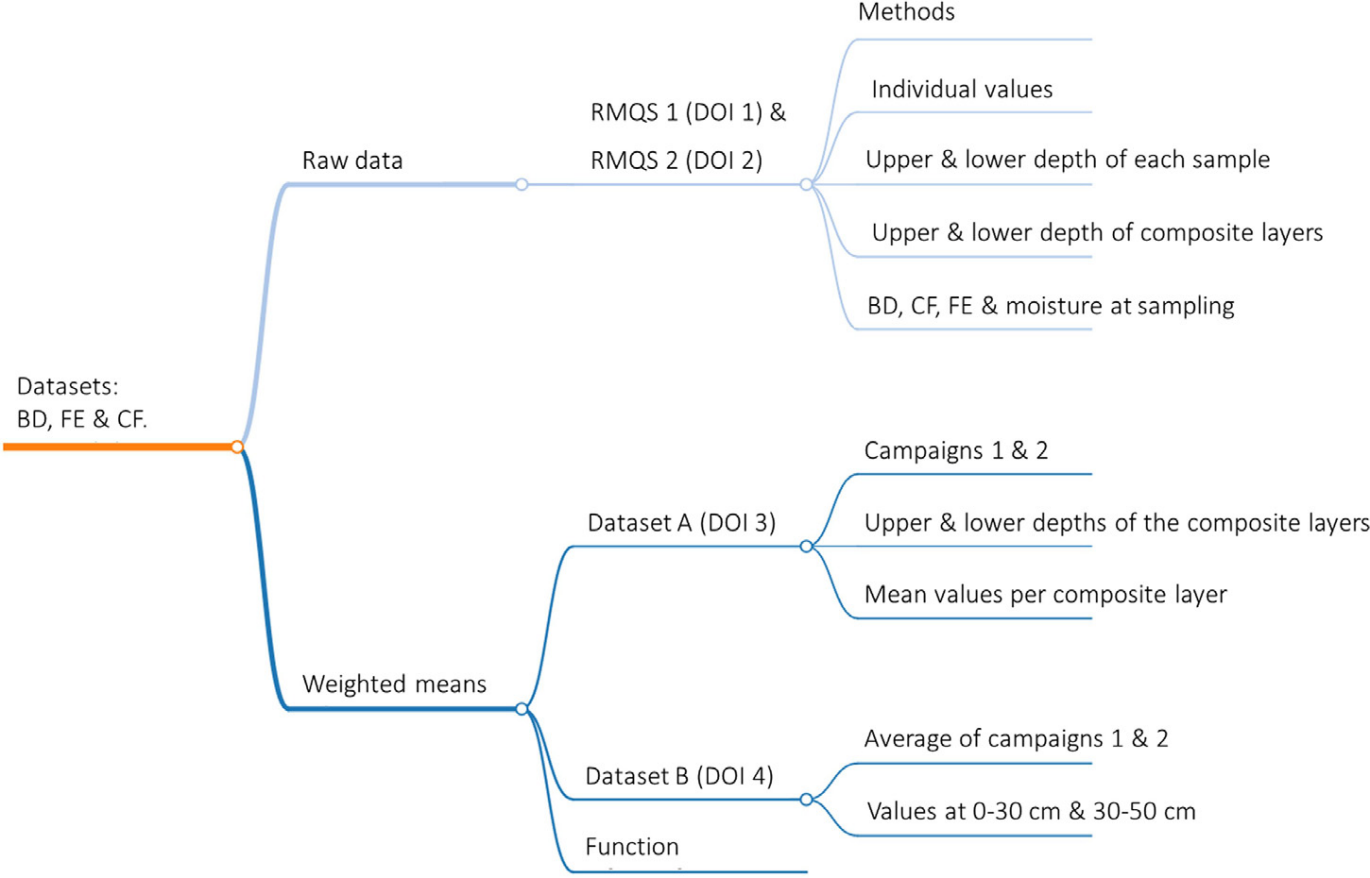
Structure of the project repository. Raw data of campaign 1 & 2 are needed to calculate datasets A and B. Dataset A includes weighted means of bulk density, coarse fragment contents, and fine earth in topsoil and subsoil in campaign 1 and 2. Dataset B contains the average values of campaigns 1 and 2 of bulk density, coarse fragment contents, and fine earth at 0–30 cm and 30–50 cm. In addition, the project repository includes the R script of the function used to calculate the weighted average for a specific soil layer.

Four datasets are provided (Fig. 1). The first dataset (file RMQS1_bulk_density_raw_data.tab) contains the raw data with the results of the individual soil physical samples for campaign 1. The second dataset (file Raw_data_rmqs_2.tab) contains the results of the individual soil physical samples for campaign 2 (see below for details on both campaigns). The third dataset (file Dataset_A.tab) provides the weighted average of BD, fine earth (FE), and CF contents for the topsoil and subsoil in the two campaigns. These weighted mean values are calculated in way that they match the thickness of the composite layers of the RMQS program for each campaign in order to compute stocks using the chemical analyses (see details in experimental design, materials and methods section). The fourth dataset (file Dataset_B.tab) includes the average values of BD, CF, and fine earth (FE) mass per total volume from both campaigns, calculated separately for the 0–30 cm and 30–50 cm layers. The data of the second campaign comprises the year 2016 until 2022.

### 3.1 Raw data

The raw data of the first and second campaigns are made available separately. The raw data of the first campaign can be found through a DOI and contains all the physicochemical properties measured in the first RMQS campaign. While the raw data of the second campaign is provided in a dataset exclusively dedicated to the soil physical data of campaign 2. The data structure of both datasets, containing the raw data for the respective campaign, is consistent. The two datasets have information about the campaign, the cell number within the RMQS grid, identification of each sampling site within a cell, the spatial coordinates, the sampling date, the identifier of the soil layer of the composite sample, land use categories, the inferior and upper limits of each individual sample, the upper and lower limits of the layer which the individual samples are attached to, the methods employed, and the values of BD, CF, and FE. More details can be found in the readme file accompanying each dataset.

### 3.2 Dataset A

**Fig. 2:**
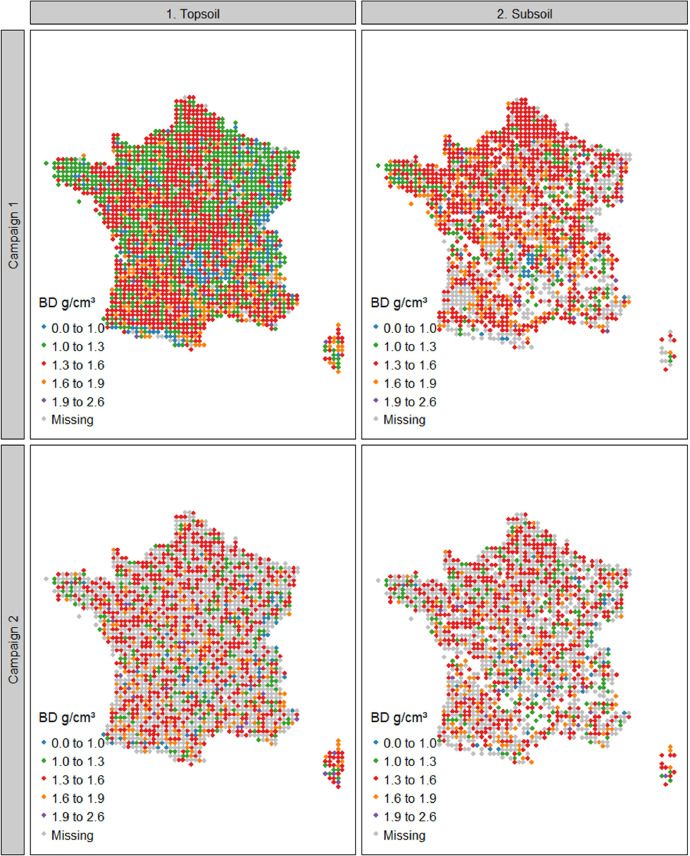
Bulk density in topsoil and subsoil in France in campaigns 1 and 2 of the French Soil Quality Monitoring Network (RMQS). Blank spaces represent sites with no developed soil. This figure was obtained using dataset A. Grey points represent sites with no soil physical data or incomplete data.

**Fig. 3:**
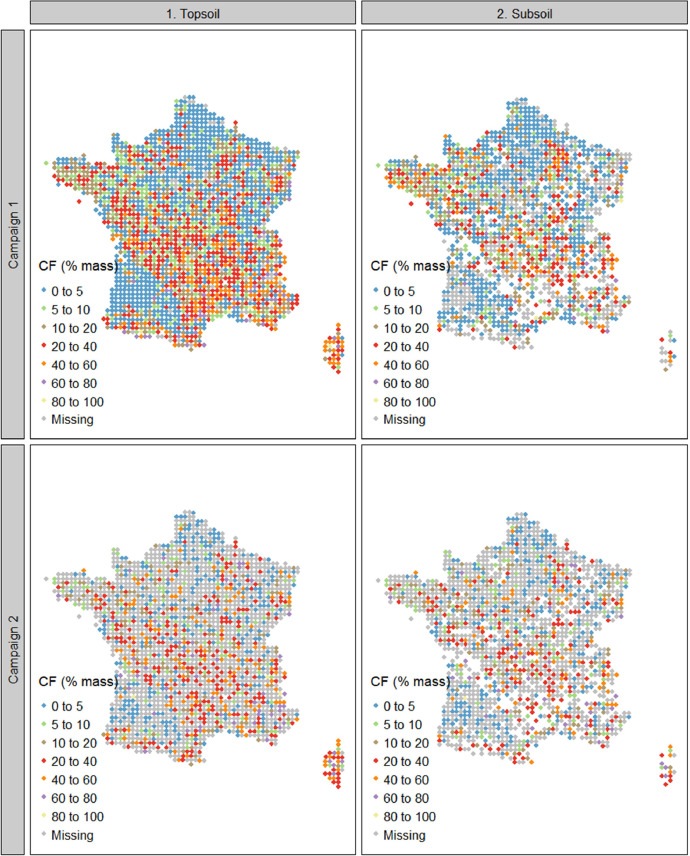
Coarse fragment content (expressed as mass percentage) in France in topsoil and subsoil in campaigns 1 and 2 of the French Soil Quality Monitoring Network (RMQS). Blank spaces represent sites with no developed soil. Grey points represent sites with missing or incomplete coarse fragment content data. This figure was obtained using dataset A.

**Fig. 4:**
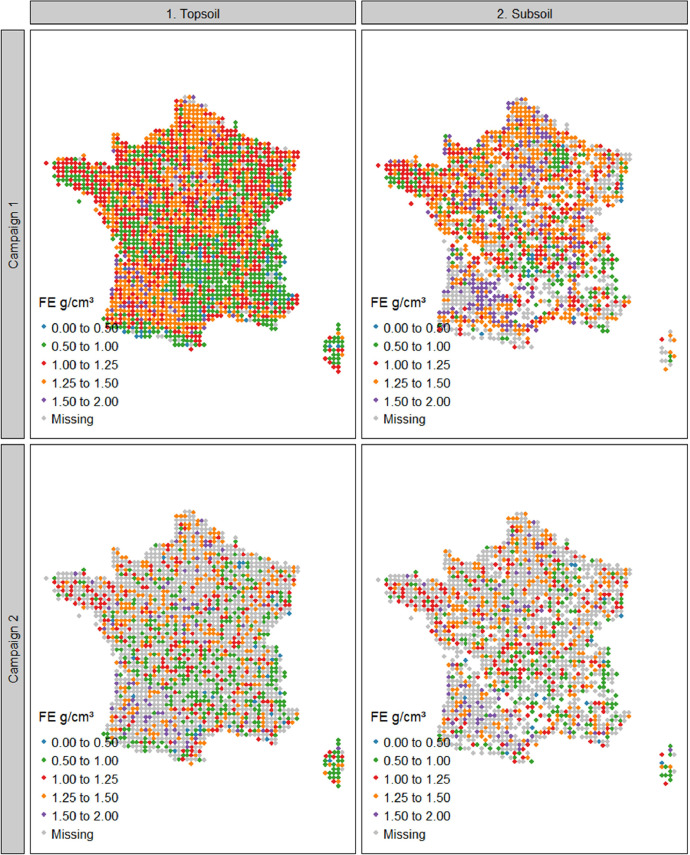
Fine earth density in topsoil and subsoil in France in campaigns 1 and 2 of the French Soil Quality Monitoring Network (RMQS). Blank spaces represent sites with no developed soil. Grey points represent sites with missing or incomplete fine earth data. This figure was obtained using dataset A

The summary statistics of Dataset A are shown in Table 1 and the spatial representation in Fig. 2, Fig. 3, Fig. 4. For campaign 1, some sites were not sampled due to inaccessibility or absence of sampling soil in the given area (e.g. military lands, mountainous rocky soils, strongly-anthropized area, steep and unsafe access; Fig. 2). For both campaigns there were more sites with data for the topsoil layer than for the subsoil (Figs. 2–4). One reason is that for most of the forest sites, during the first campaign, the BD sampling was conducted only for the topsoil due to time and budget constraints. Another reason is that a rather large proportion of sites simply did not have a developed subsoil layer (Fig. 3).

**Table 1:** Mean values, standard deviation, minimal values, first, second and third quantiles, maximal values and number of observations of bulk density, fine earth and coarse fragment content in topsoil and subsoil in campaigns 1 and 2 of the French Soil Quality Monitoring Network (RMQS).

Bulk density (g cm^−3^)									
Campaign	Layer	Mean	Sd	Min	Q1	Q2	Q3	Max	n
1	1. Topsoil	1.31	0.23	0.19	1.18	1.32	1.45	2.1	2149
1	2. Subsoil	1.46	0.21	0.2	1.36	1.47	1.57	2.34	1365
2	1. Topsoil	1.28	0.23	0.33	1.15	1.29	1.42	2.28	1240
2	2. Subsoil	1.45	0.22	0.34	1.34	1.46	1.56	2.56	1006

Coarse fragments (% mass or g. 100 g^−1^)									
Campaign	Layer	Mean	Sd	Min	Q1	Q2	Q3	Max	n
1	1. Topsoil	16.62	17.74	0	1.97	10.01	27.65	88.75	2135
1	2. Subsoil	13.5	16.59	0	0.9	6.51	20.55	88.37	1353
2	1. Topsoil	20.79	18.01	0	5.09	16.74	32.83	92.04	970
2	2. Subsoil	18.3	18.94	0	2.3	11.46	29.85	83.68	810

Fine earth mass per total volume (g cm^−3^)									
Campaign	Layer	Mean	Sd	Min	Q1	Q2	Q3	Max	n
1	1. Topsoil	1.07	0.27	0.18	0.9	1.1	1.29	1.76	2135
1	2. Subsoil	1.25	0.27	0.2	1.1	1.31	1.45	1.81	1353
2	1. Topsoil	1	0.26	0.14	0.81	1.02	1.2	1.67	970
2	2. Subsoil	1.17	0.29	0.33	0.97	1.23	1.41	1.92	810

### 3.3 Dataset B

**Fig. 5:**
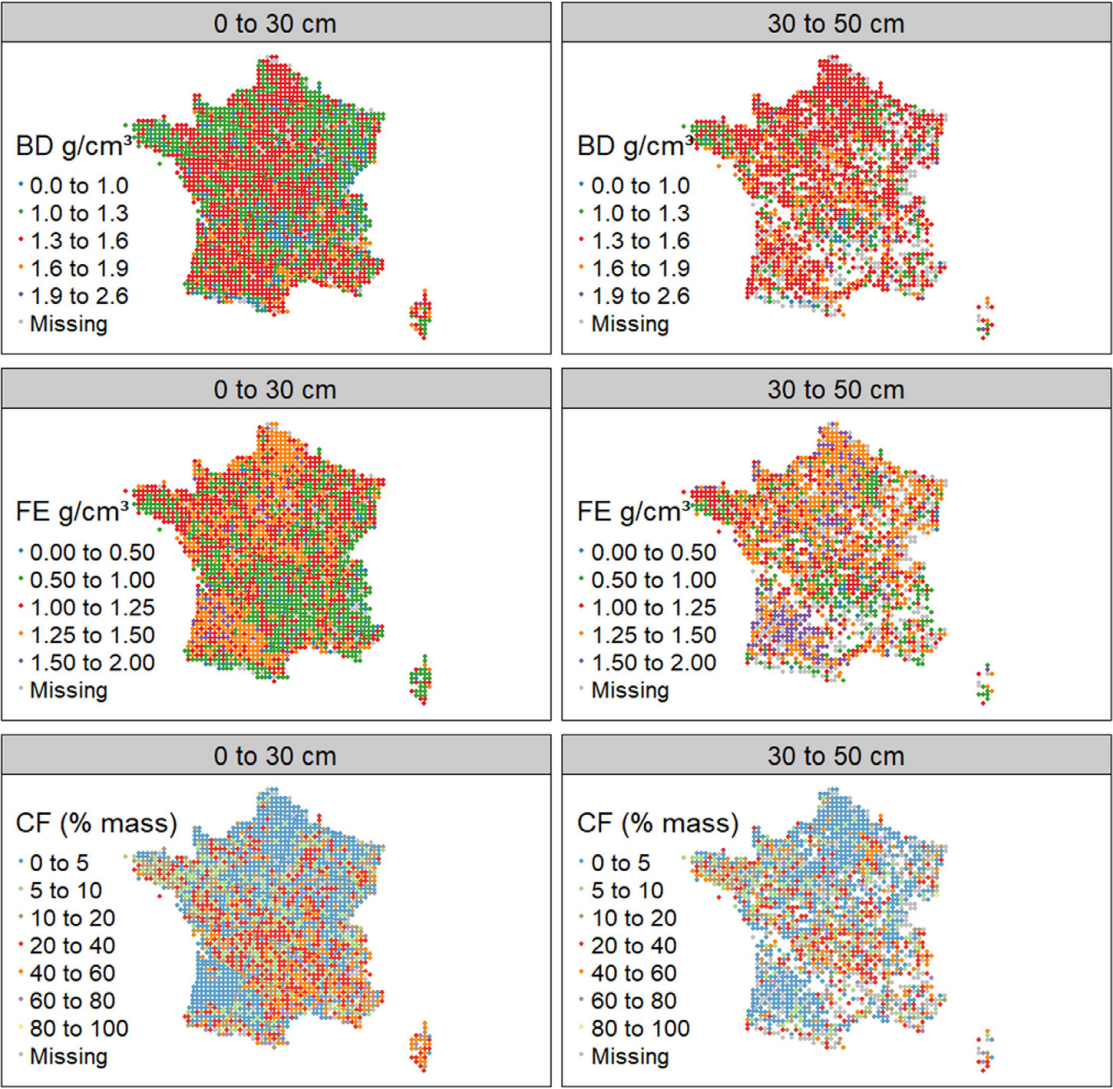
Average bulk density, fine earth, and coarse fragment content of campaigns 1 and 2 of the French Soil Quality Monitoring Network (RMQS) at 0–30 cm and 30–50 cm. Upper panel: bulk density at 0–30 cm and 30–50 cm. Middle panel: fine earth at 0–30 cm and 30–50 cm. Lower panel: coarse fragment content at 0–30 cm and 30–50 cm. Blank spaces represent sites with no formed soil. This figure was obtained using dataset B. Grey points represent sites with missing or incomplete data.

The summary statistics of Dataset B are shown in Table 2 and the spatial representation in Fig. 5.

**Table 2:** Mean values, standard deviation, minimal values, first, second and third quantiles, maximal values and number of observations of bulk density, fine earth and coarse fragment content at 0–30 cm and 30–50 cm. The values are the average of campaigns 1 and 2 of the French Soil Quality Monitoring Network (RMQS). This table was produced using Dataset B.

Bulk density (g cm^−3^)								
Layer	Mean	Sd	Min	Q1	Q2	Q3	Max	n
0 to 30 cm	1.30	0.22	0.19	1.19	1.32	1.44	2.1	2147
30 to 50 cm	1.45	0.21	0.2	1.35	1.47	1.57	2.56	1597

Coarse fragments (% mass or g. 100 g^−1^)								
Layer	Mean	Sd	Min	Q1	Q2	Q3	Max	n
0 to 30 cm	16.6	17.61	0	1.69	10.1	27.89	90.17	2147
30 to 50 cm	14.82	17.66	0	0.94	7.49	23.70	86.26	1597

Fine earth mass per total volume (g cm^−3^)								
Layer	Mean	Sd	Min	Q1	Q2	Q3	Max	n
0 to 30 cm	1.07	0.27	0.17	0.9	1.1	1.28	1.69	2147
30 to 50 cm	1.22	0.28	0.2	1.06	1.3	1.44	1.81	1597

## 4 Experimental Design, Materials and Methods

### 4.1 Soil sampling sites

The French soil quality monitoring network (RMQS) is comprised of approximately 2170 locations regularly distributed across the mainland territory, following a systematic random sampling grid with a resolution of 16 km [12]. The sampling sites were initially chosen to be at the central point of each grid cell. However, in instances where accessibility was a challenge (in the event, for example, of refusal by the land owner), alternative sites within a 1-km radius of the cell center were selected. The first RMQS campaign was conducted between 2000 and 2009, and the second campaign is currently ongoing, scheduled from 2016 to 2027. The sampling during the first campaign was carried out by zone, successively sampling departments and/or regions according to the arranged contracts. The strategy for the sampling process in the second campaign was completely revised to become globally annualized: the sites sampled each year are now distributed geographically in a regular way throughout the national territory.

**Fig. 6:**
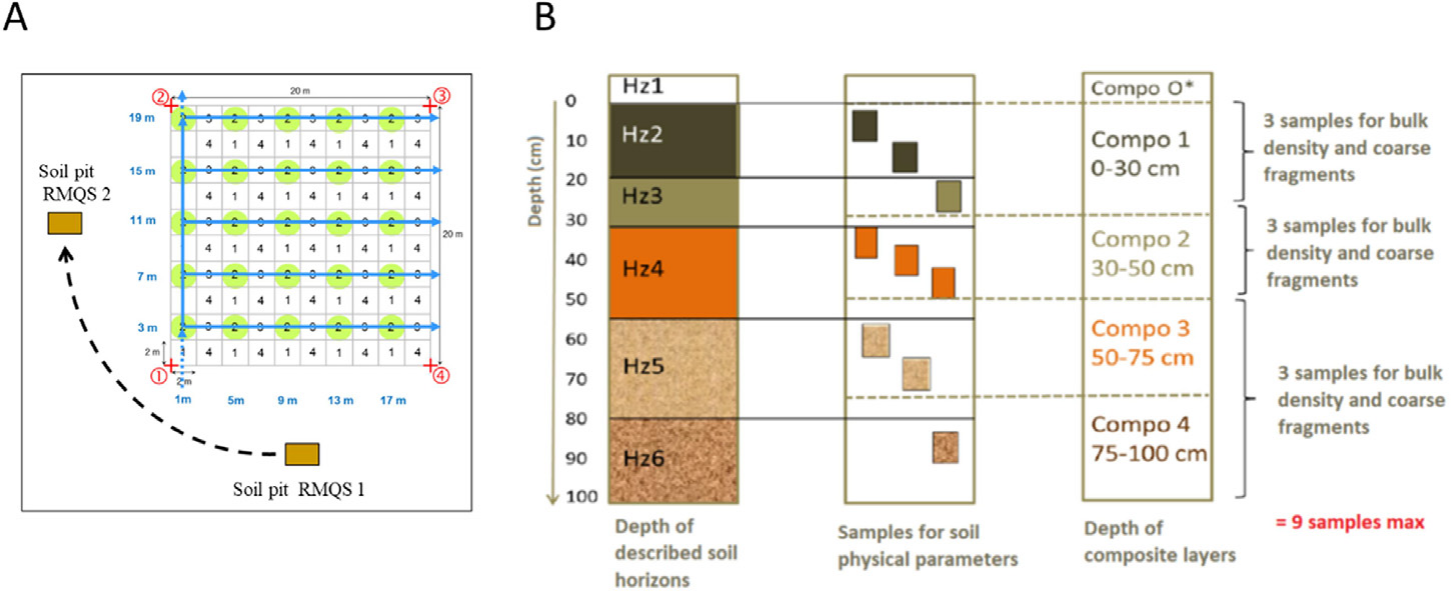
A) Systematic unaligned grid used for composite sampling, and two soil pits used for soil profile description. The position of the soil pit was changed from the southern side of the grid in the first campaign to the west side in the second campaign. B) Right: representation of a soil profile described in the soil pits for the second campaign. For the first campaign, physical measurements were done up to 50 cm only. Center: distribution of the soil physical samples within the soil profile. Right: Composite layers sampled in the grid. Taken from Jolivet et al. [12].

At each of these sampling sites, two types of samples were obtained. First, a composite sample was obtained by aggregating 25 soil cores following an unaligned sampling scheme using a 20-m by 20-m grid (Fig. 6A) divided into 100 quadrats of 2 m by 2 m. The quadrats with the number 1 were used for sampling in the first campaign, the ones with number 2 in the second campaign, while the ones with numbers 3 and 4 will be used in the third and fourth campaigns, respectively (Fig. 6A). Adjacent to the 20-m by 20-m grid, a soil pit was dug to a depth of 1 m when possible or until parent material for profile description and for the second type of samples, i.e. volumetric sampling, specifically for BD measurement and CF content assessment. The location of the soil pit was changed from the southern side of the grid during the first campaign to the western side in the second campaign, as illustrated in Fig. 6A. This resulted in a distance of approximately 20 m between the two soil pits dug at each campaign.

Three soil physical samples were collected from the topsoil layer (0–30 cm) and an additional three from the subsoil layer, totaling three samples for each composite layer up to 50 cm (when soil profile was deep enough) for both campaigns (Fig. 6B). In the second campaign, the three soil physical samples per composite layer were specifically confined to pedogenetic soil horizons, ensuring that samples did not span across different horizons.

Composite sampling was conducted within two soil layers: topsoil at 0–30 cm and subsoil at 30–50 cm. In some cases, there was a gap between the lower limit of the topsoil layer and the upper limit of the subsoil layer due to the presence of plowing horizons detected in the profile description conducted in the soil pit, especially in croplands. Thus, the inferior limit of the topsoil layer and the upper limit of the subsoil layer was not always 30 cm.

The composite samples were used for analyzing various soil chemical parameters, including soil organic carbon, pH, soil nutrients, heavy metals, and more. The current work does not include the data obtained from the composite samples. However, this reference is made to the composite layers because the BD and CF values are calculated to match the thickness of each of the layers for later computation of stocks of elements, for instance, whose concentration is done on the composite samples.

### 4.2 Methods for bulk density measurement

The methods employed to determine soil BD were the use of the ring or cylinder and excavation methods, using either water or sand (as per NF ISO 11272, 2017; NF X31-503, 1992). The cylinder or ring method involved the use of a 500 cm^3^ ring, which was either applied at the soil surface or within the walls of the soil pit, particularly in cases involving deeper parts of the soil profile. In cases where the cylinder was employed for deeper layers, a horizontal surface was carefully prepared using a trowel, followed by the insertion of the cylinder with the aid of a rubber hammer.

On the other hand, the excavation method was adopted for soils characterized by high contents of rocks, gravel, roots, or in terrains with a sloping topography. When used in subsurface layers, a flat surface was created with a trowel, and a metallic rounded template with an inner diameter of approximately 13 cm was used to ensure the surface's evenness. Subsequently, this template served as a guide for excavation. A cavity, ranging from about 1000 to 1500 cm^3^ in volume, was carefully dug with a trowel, and all the soil was collected in a plastic bag. Another plastic bag was then fitted against the cavityʼs walls, and its volume was estimated by filling it with a known quantity of either water or calibrated sand. The single difference between excavation using water and sand lies in the fact that the latter can be applied when achieving a completely flat surface is not possible.

The collected soil samples were transported to the European Conservatory of Soil Samples in Orléans, France, where the moist mass was recorded. After weighing the moist mass, the samples were subjected to oven-drying at 105 °C, followed by weighing and sieving through a 2 mm mesh. The weight of the coarse fragments (> 2 mm), after oven-drying at 105 °C, was also recorded. In some cases of the second campaign, the CF were not weighed. The decision of not weighing the CF was adopted when the cylinder method was used in both campaigns and when the mass of each of the three samples the considered layer was < 50 g in the first campaign.

### 4.3 Fine earth, bulk density, and coarse fragment fraction

The calculation of bulk density for each sample involved dividing the total soil mass (*m_tot_*), comprising dried fine earth mass (*m_fine_*) and coarse fragment mass (*m_coarse_*), by the volume of the sample (*V_tot_*):

(1) $$m_{tot}=m_{fine}+m_{coarse}$$

(2) $$BD=\frac{m_{tot}}{V_{tot}}$$

The density of the fine earth (FE) was calculated as the ratio of *m_fine_* divided by *V_tot_*:

(3) $$FE=\frac{m_{fine}}{V_{tot}}$$

To avoid confusion, please note that the definition of FE is different from the bulk density of the fine soil (BD_fine soil_) as defined in Eq. (3) of the article by Poeplau et al. [11].

The coarse fragment fraction (*coarse_mass_*) was calculated as the ratio between the mass of the coarse fragments and *m_tot_*:

(4) $$coarse_{mass}=\frac{m_{coarse}}{m_{tot}}$$

### 4.4 Data treatment of physical measurements

**Fig. 7:**
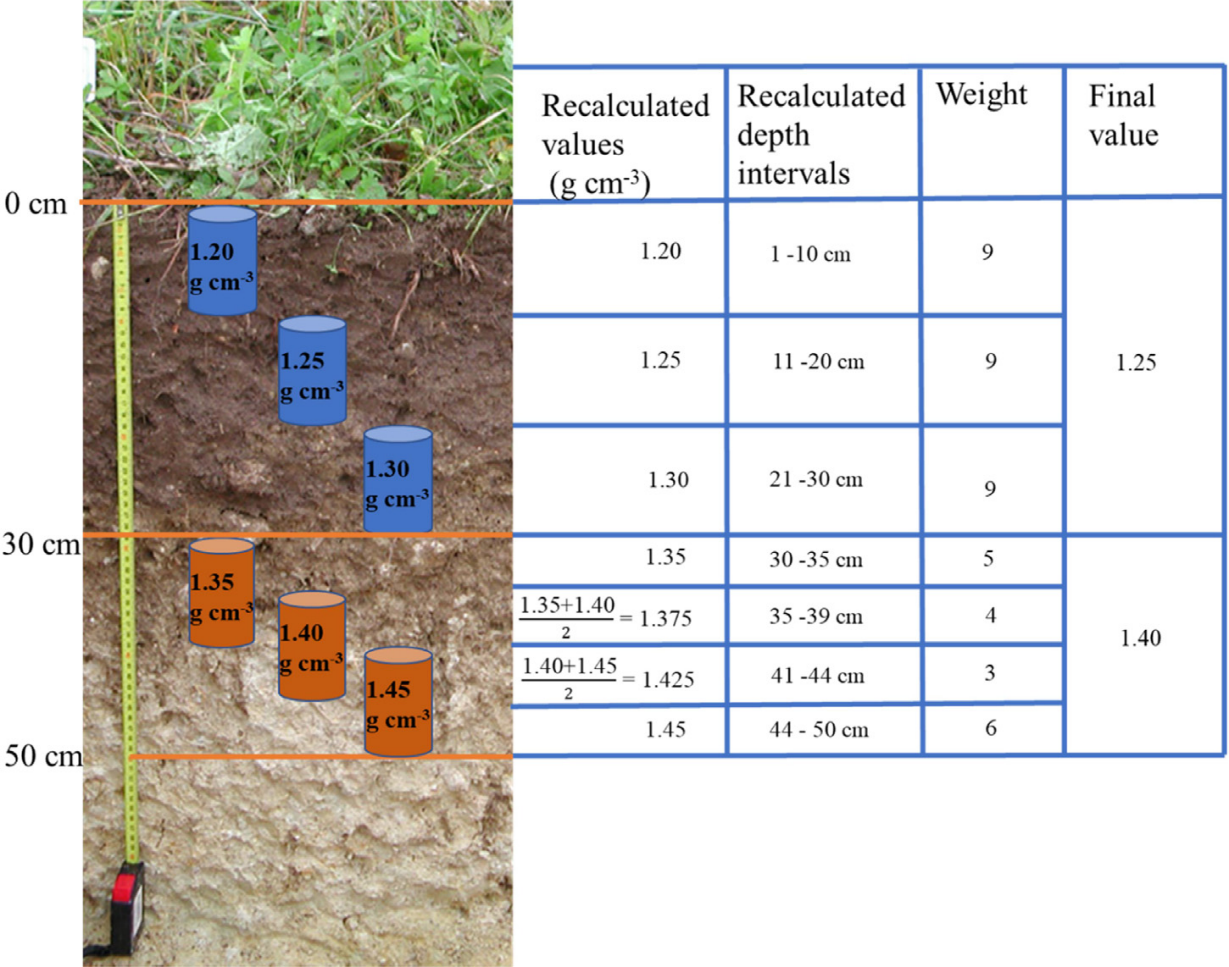
Diagram of the calculation of the weighted average of the soil physical data. The depth intervals are recalculated when there is an overlap of individual samples. Next a weight is attributed to the new depth intervals to allow the calculation of the values for the topsoil and subsoil layers.

In dataset A, depth-weighted averages of BD, FE, and *coarse_mass_* were calculated for topsoil and subsoil for both campaign 1 and campaign 2. The thickness of both layers corresponds to the composite layers sampled from the 20-m by 20-m grid. In most of the cases the thickness of topsoil was 30 cm, and 20 cm for the subsoil. As mentioned above, in some cases the thickness of both layers was different. Thus, for dataset A we refer to topsoil and subsoil and avoid referring to a general value for the upper and lower limits of each composite layer. For the calculation of the weighted averages, we redefined the depth intervals in cases where there was an overlap of individual soil physical samples collected from the same depth ranges (Fig. 7). This adjustment was made to prevent an over-representation of depth intervals covered by multiple soil samples. For each of these newly defined depth intervals, we computed an average, and subsequently, the weighted mean was calculated, with weights based on the length of each respective depth interval (Fig. 7).

For dataset B, we redefined the depth intervals from 0 to 50 cm using the individual samples of campaign 1 and 2. Next, the weighted averages were calculated for the layers 0–30 cm and 30–50 cm. The R code of the function that was used to calculate the weighted averages is given in the project repository, as well as the R scripts to calculate datasets A and B.

## Limitations

The mass (and volume) of living roots is neglected in the current datasets. As discussed by Poeplau et al. [11], roots are considered as part of plant biomass and not as SOC. In addition, CF ≥ 20 cm are not measured in the RMQS program, hence this is a factor of overestimation of the fine earth mass in some cases.

## Ethics Statement

The present data do not involve human subjects, animal experiments, or any data collected from social media platforms.

## CRediT authorship contribution statement

**Jose-Luis Munera-Echeverri:** Conceptualization, Methodology, Software, Data curation, Visualization, Writing – original draft, Writing – review & editing. **Line Boulonne:** Conceptualization, Methodology, Software, Data curation, Writing – review & editing. **Nicolas P.A. Saby:** Conceptualization, Methodology, Software, Writing – review & editing. **Dominique Arrouays:** Conceptualization, Methodology, Writing – review & editing. **Benoît Bertouy:** Software. **Eva Lacarce:** Methodology. **Floriane Serré:** Methodology. **Benoit Toutain:** Methodology. **Florent Millet:** Methodology. **Thomas Loiseau:** Methodology. **Manuel Martin:** Conceptualization, Methodology, Software, Writing – review & editing, Funding acquisition.

## Data Availability

Raw bulk density and coarse fragment data of the first campaign of the French Soil Quality Monitoring Network. (Original data) (GisSol Dataverse) Raw bulk density and coarse fragment data of the second campaign of the French Soil Quality Monitoring Network (Original data) (GisSol Dataverse) Weighted average of bulk density, coarse fragment by layer of the first and second campaign of the French Soil Quality Monitoring Network. (Original data) (GisSol Dataverse) Average values of bulk density and coarse fragment at 0-30 cm and 30-50 cm of the first and second campaigns of the French Soil Quality Monitoring Network. (Original data) (GisSol Dataverse)
